# Biological and Clinical Implications of Comorbidities in Parkinson’s Disease

**DOI:** 10.3389/fnagi.2017.00394

**Published:** 2017-12-04

**Authors:** Jose A. Santiago, Virginie Bottero, Judith A. Potashkin

**Affiliations:** Department of Cellular and Molecular Pharmacology, The Chicago Medical School, Rosalind Franklin University of Medicine and Science, North Chicago, IL, United States

**Keywords:** anemia, cancer, comorbidities, depression, diabetes, Parkinson’s disease, personalized medicine

## Abstract

A wide spectrum of comorbidities has been associated with Parkinson’s disease (PD), a progressive neurodegenerative disease that affects more than seven million people worldwide. Emerging evidence indicates that chronic diseases including diabetes, depression, anemia and cancer may be implicated in the pathogenesis and progression of PD. Recent epidemiological studies suggest that some of these comorbidities may increase the risk of PD and precede the onset of motor symptoms. Further, drugs to treat diabetes and cancer have elicited neuroprotective effects in PD models. Nonetheless, the mechanisms underlying the occurrence of these comorbidities remain elusive. Herein, we discuss the biological and clinical implications of comorbidities in the pathogenesis, progression, and clinical management, with an emphasis on personalized medicine applications for PD.

## Parkinson’s Disease: Beyond Motor Symptoms

Parkinson’s disease (PD) is an incurable neurodegenerative disease affecting 7–10 million people worldwide^1^. PD is clinically categorized as a movement disorder with prominent motor symptoms, which include tremors, rigidity and bradykinesia (Hoehn and Yahr, 1967; Poewe et al., 2017). Motor symptoms usually appear late in the disease process as a result of dopaminergic cell death and accumulation of alpha synuclein (SNCA), a major constituent of Lewy bodies and a pathological hallmark of PD (Samii et al., 2004; Venda et al., 2010). Current therapies for PD confer symptomatic relief but to date, there is no treatment available to halt or slow the progression of the disease. The lack of fully validated biomarkers to detect patients in the early stages of the disease continues to be a major limitation in the design and outcome of clinical trials testing potential drugs and/or neuroprotective agents.

**Figure 1:**
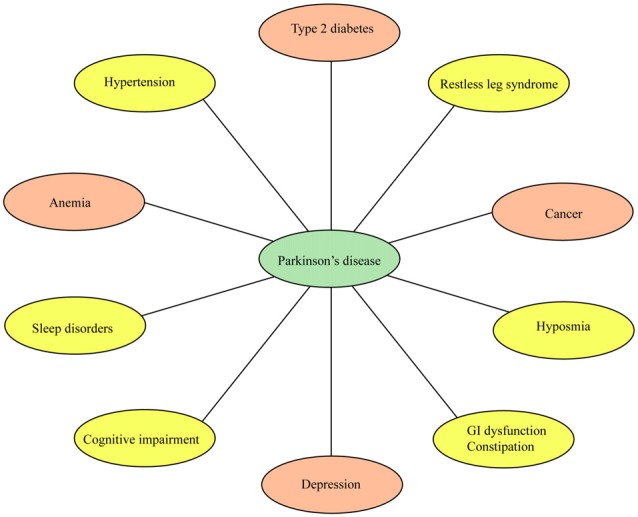
Non-motor conditions and comorbidities associated with Parkinson’s disease (PD). Non-motor conditions and comorbidities have a detrimental impact in the quality of life and clinical status of PD patients. Some of these conditions may precede the onset of PD. Drugs to treat type 2 diabetes, depression, anemia and cancer are currently being tested in clinical trials for PD (orange ovals).

Although PD is primarily categorized as a movement disorder, a wide range of non-motor conditions are increasingly being recognized as early features of the disease (Park and Stacy, 2009; Visanji and Marras, 2015; Papagno and Trojano, 2017; Schapira et al., 2017; Trojano and Papagno, 2017; Figure 1). Non-motor symptoms frequently reported in PD patients include cognitive impairment, dementia, constipation, fatigue, hyposmia, restless legs syndrome (RLS), and sleep behavior disorder (RBD), urinary problems, drooling and hallucinations (Fenelon et al., 2000; Park and Stacy, 2009; Burghaus et al., 2012; Goldman et al., 2014; Zhang and Zhang, 2015; Karakoc et al., 2016; Papagno and Trojano, 2017; Schapira et al., 2017; Trojano and Papagno, 2017). Non-motor complications have a detrimental impact in the quality of life and health status of PD patients. Cognitive impairment and dementia, for example, are disabling conditions that need special attention by clinicians as these may have detrimental effects in patients. The presence of non-motor symptoms is evident in early stage drug-naïve PD patients (Goldman et al., 2014) and in subjects without dopamine deficits (Sprenger et al., 2015) thus suggesting these conditions may be precursors of neurodegeneration. The non-motor symptoms in drug-naïve PD patients have been under recent investigations (Kwon et al., 2014; Spalletta et al., 2014; Erro et al., 2015).

Several studies have investigated the extent and the impact of comorbidities in PD. For example, a population-based study identified significant comorbid conditions including bone fractures, cancer, dementia, diabetes and stroke in PD patients (Leibson et al., 2006). A longitudinal study found hypertension and diabetes as the most frequent comorbidities in PD patients (Santos García et al., 2017). Recent studies have also shown that chronic diseases like anemia and cancer may be implicated in the pathogenesis of PD. Depression may appear decades before the onset of PD. Nonetheless, very little is known about the mechanisms by which some of these comorbidities may be implicated in the pathogenesis and progression of PD. More importantly, recent studies investigating the connection between PD and comorbid conditions have uncovered novel therapeutic targets and diagnostic biomarkers. In fact, drugs to treat diabetic patients are currently under evaluation in clinical trials for PD. Thus, understanding the molecular mechanisms underlying the association between PD and comorbidities is expected to advance personalized medicine for PD patients.

In this review article, we discuss the biological and clinical implications of the most common chronic comorbidities in sporadic PD. A search strategy was performed in Pubmed to identify relevant articles published in English on or before October 26, 2017. The keywords used during the searches included “PD” with the following words: “motor symptoms”, “non-motor symptoms”, “comorbidities”, “biomarkers”, therapeutics” “cognitive symptoms”, “diabetes”, “depression”, “anemia” and “cancer” located within the title and/or abstract. For this study, we chose to focus on comorbidities for which therapeutic agents have been tested on PD patients.

## Diabetes and Parkinson’s Disease

Diabetes is a growing public health concern affecting approximately 415 million people worldwide. According to the International Diabetes Federation, the incidence of diabetes is expected to rise to 642 million by 2040 making diabetes one of the most prevalent chronic diseases globally. There are three types of diabetes, type 1, type 2 and gestational diabetes. The most common form of diabetes is type 2 diabetes characterized by pancreatic beta cell dysfunction leading to insulin resistance and high blood glucose levels. Diabetes has been implicated in several neurodegenerative diseases including, Alzheimer’s disease (AD; Akter et al., 2011), Amyotrophic lateral sclerosis (ALS; Kioumourtzoglou et al., 2015) and PD (Santiago and Potashkin, 2013b). The increasing evidence that implicates diabetes as the root cause for some of the most devastating neurodegenerative diseases heightens the urgency to better understand the mechanisms by which diabetes leads to neurodegeneration.

An association between PD and diabetes was first noted more than two decades ago (Sandyk, 1993). Since then, a growing number of epidemiological, clinical and molecular studies have investigated a potential link between PD and diabetes. Most of the epidemiological studies from diverse populations suggest that diabetes is associated with an increased risk of PD (Table 1). A large population-based prospective study found an increased risk of PD among Finnish men and women (Hu et al., 2007). During a mean follow-up period of 18 years, 324 out of 591 men and 309 out of 507 women with diabetes developed PD. Likewise, a prospective study in the United States identified a 40% increased risk of PD among cases of self-reported diabetes (Xu et al., 2011). Another prospective study found that men with diabetes had a higher risk of PD compared with non-diabetic men (Driver et al., 2008). Case-control studies have found a positive association between both diseases in different countries including Denmark, China, and Taiwan (Schernhammer et al., 2011; Sun et al., 2012; Wahlqvist et al., 2012). These findings are supported by recent studies investigating the relationship between PD and diabetes. For instance, a meta-analysis including seven population-based cohort studies representing 17,61,932 individuals indicated that diabetes is associated with a 38% increased risk of PD (Yue et al., 2016). More recently, a Taiwanese study consisting of 36,294 patients with newly diagnosed diabetes found an incidence PD rate of 1.36 fold higher among diabetes compared to non-diabetic patients (Yang et al., 2017). This population-based study found a 23% increased risk of PD among all diabetic patients after adjusting for age, gender, occupation, insurance premium and other comorbidities. Despite these evidences, there are some studies that have found a negative association. For example, a large meta-analysis including 14 case-control studies reported a negative association between PD and diabetes (Lu et al., 2014). Yet, the same study reported a higher percentage of PD patients had a diagnosis of diabetes, 2.6% compared to the 1.6% of the control group. Conflicting results between studies may be attributed to differences in the study design (case-control vs. prospective), PD and diabetes cases ascertainment, misdiagnosis of PD cases, and failure to control for medication effects on patients.

**Table 1:** Studies investigating the association between diabetes and Parkinson’s disease.

Study	Study design	Main results
Hu et al. (2007)	Prospective, cohort study, Finnish population	Diabetes is associated with a higher than 50% increased risk of PD in men and women.
Driver et al. (2008)	Prospective, cohort study, USA	Diabetes is associated with an increased risk of PD in men.
Xu et al. (2011)	Prospective, cohort study, USA	Diabetes is associated with a 40% increased risk of PD.
Schernhammer et al. (2011)	Case-control, Danish population	Diabetes is associated with a 36% increased risk of PD, specially younger onset PD.
Bosco et al. (2012)	Case-control, Italian population	PD patients with dementia are two times more likely to have insulin resistance than patients with PD.
Sun et al. (2012)	Retrospective, case-control, Chinese population	Diabetes is associated with an increased risk of PD. Association is stronger in women and younger patients.
Wahlqvist et al. (2012)	Case-control, Taiwanese	Treatment with metformin-sulfonylurea is associated with a reduced risk of PD.
Cereda et al. (2012)	Retrospective, case-control	Onset of diabetes before onset of PD is associated with more severe symptoms and reduced efficacy of levodopa therapy.
Kotagal et al. (2013)	Case-control	PD patients with diabetes exhibit greater postural instability and gait difficulty.
Santiago and Potashkin (2013a),	Network analysis	PD and diabetes share molecular networks.
Santiago et al. (2014)		
Bohnen et al. (2014)	Case-control	Diabetes is associated with severe cognitive impairment in PD.
Yue et al. (2016)	Meta-analysis	Diabetes is associated with a 38% increased risk of PD.
Yang et al. (2017)	Retrospective, case-control, Chinese	Diabetes is associated with a 23% increased risk of PD, especially in females.

Diabetes has been associated with more severe motor symptoms and accelerated disease progression in PD patients. For example, patients with diabetes who developed PD had higher Unified Parkinson Disease Rating Scale (UPDRS) motor scores and more severe Hoehn and Yahr staging (Cereda et al., 2012). In addition to disease progression, diabetes has been associated with specific symptoms in PD patients including postural instability, gait difficulty, dementia and cognitive impairment (Bosco et al., 2012; Kotagal et al., 2013; Bohnen et al., 2014). The authors suggested that these associations are likely mediated through other mechanisms other than dopaminergic cell death. Future mechanistic studies will be crucial to better understand the link between both diseases.

The precise mechanism by which diabetes is linked to PD remains unknown but several hypotheses have been postulated (Geng et al., 2011; Aviles-Olmos et al., 2013; Santiago and Potashkin, 2013b, 2014b). It is well documented that PD and diabetes share remarkably similar dysregulated pathways. For example, inflammation, mitochondrial dysfunction, endoplasmic reticulum stress, autophagy and impaired insulin signaling are among some of the shared mechanisms between both chronic diseases (Santiago and Potashkin, 2013b). We hypothesized that genetic susceptibility, lifestyle choices and exposure to toxic environmental factors may lead to the disruption in these pathways and ultimately trigger diabetes and/or neurodegeneration. Inflammation and impaired insulin signaling, for example, are currently being targeted for therapeutic intervention (Santiago and Potashkin, 2014b). It remains unclear, however, whether inflammation and/or impaired insulin signaling are causative factors, precursors or a consequence of the neurodegenerative process (Aviles-Olmos et al., 2013; Santiago and Potashkin, 2013b). Interestingly, early stage drug-naïve PD patients and PD patients with mild cognitive impairment exhibited blood glucose levels characteristic of pre-diabetes (≥100 mg/dL) thus suggesting that impaired glucose metabolism is an early event in PD (Santiago and Potashkin, 2015a). In the context of inflammation, it has been demonstrated that blood myeloid dendritic cell frequency declines in idiopathic PD patients and is associated with motor symptoms severity (Ciaramella et al., 2013). The authors have proposed that blood dendritic cells may play an essential role in the pathogenesis and progression of PD and that monitoring dendritic cell frequency in blood could offer a clinical tool for PD diagnosis and treatment (Bossu et al., 2015).

In addition to sharing disruption in common dysregulated pathways, PD and diabetes share some of the same genetic and environmental risk factors. For example, genetic variants in *AKT*, involved in the regulation of cell survival and apoptosis, have been associated with PD and diabetes (Xiromerisiou et al., 2008; Li et al., 2016). Environmental exposure to heavy metals and pesticides has been associated with an increased risk of PD (Willis et al., 2010) and diabetes (Chen et al., 2009; Alonso-Magdalena et al., 2011).

Drugs used to treat diabetes patients have shown promise in improving motor symptoms in PD patients. One notable therapeutic target is the glucagon-like peptide 1 (GLP-1) receptor. GLP-1 mimetics including, exenatide, liraglutide and lixisenatide have shown efficacy in alleviating some of the motor symptoms in PD patients. Among these drugs, PD patients treated with exenatide showed improvement in motor symptoms and cognitive domains after 1 year of treatment (Aviles-Olmos et al., 2014). More recently, a double-blinded and placebo-controlled trial showed that treatment with exenatide improved motor symptoms in PD patients in an off-medication state compared with those given placebo (Athauda et al., 2017a,b). These drugs are particularly attractive due to their capacity to cross the blood brain barrier and participate in several biological pathways within the central nervous system including, neuroinflammation, mitochondrial function and brain insulin resistance (Aviles-Olmos et al., 2014). Another extensively studied therapeutic target is the peroxisome proliferation-activated receptor gamma (PPARγ). Thiazolidinediones are a class of PPAR agonist medications that are currently being investigated in PD, although with some contradictory results. For example, glitazone has been associated with a decreased incidence of PD in a retrospective cohort study in United Kingdom (Brauer et al., 2015). However, a double-blinded placebo controlled clinical trial testing pioglitazone, also a PPAR agonist, failed to modify progression in PD patients and the investigators did not recommend a larger trial (NINDS Exploratory Trials in Parkinson’s Disease (NET-PD) ZONE Investigators, 2015). Despite the negative outcome, other investigators have raised very important limitations to this study that should be taken into consideration when deciding whether to stop or continuing investigating a potential neuroprotective agent in PD (Brundin and Wyse, 2015). Firstly, the authors questioned the time period the drug was evaluated and suggest that the potential mechanism of action of pioglitazone may require a longer time to fully manifest in PD patients. In this study, PD patients with signs of disturbed glucose homeostasis were excluded. Given the strong links between PD and diabetes, it is conceivable that treatment with pioglitazone may have resulted in a positive outcome in this group of patients that were excluded. Additionally, PD patients were on other medications including rasagline or selegiline that may have masked the effects of the drug under study. Future clinical trials including drug-naïve and insulin resistance PD patients will be crucial to assess the neuroprotective effects of antidiabetic drugs.

## Depression and Parkinson’s Disease

Depression is a complex psychiatric disease affecting 6.7% of the USA population and it is recognized as the leading cause of disability worldwide^2^. Depression is considered one of the most frequent non-motor symptoms occurring in approximately 35% of PD patients (Aarsland et al., 2011) and one of the strongest predictor of quality of life (Cummings, 1992; Gallagher et al., 2010; Balestrino and Martinez-Martin, 2017). Characteristic symptoms of depression including loss of appetite, sleep disturbances, fatigue and loss of energy, are commonly observed in PD patients (Schrag et al., 2007). Several studies have shown that patients with PD suffer more frequently from depressive symptoms than other patients diagnosed with diseases with comparable disability (Nilsson et al., 2002). In fact, depression has been proposed to be a risk factor for PD. Earlier epidemiological studies showed that patients with depression have an increased risk of PD, odds ratio 2.2 (confidence interval (CI) 95% 1.7–2.8) compared to patients with other chronic conditions including osteoarthritis and diabetes (Nilsson et al., 2001, 2002). Interestingly, numerous epidemiological studies have reported an increased prevalence of depression before the clinical onset of PD supporting a causative role in the pathogenesis of PD (Shiba et al., 2000; Schuurman et al., 2002; Leentjens et al., 2003b; Fang et al., 2010; Jacob et al., 2010; Shen et al., 2013; Table 2). Several studies have established an association between PD and depression (Shiba et al., 2000; Chaudhuri et al., 2006; Pellicano et al., 2007; Postuma et al., 2012; Cooney and Stacy, 2016; Elbaz, 2016). More recently, a direct association between depression and subsequent development of PD was confirmed in a large case-control study including over 140,000 individuals with depression. Strikingly, the association between depression and PD was observed for a follow-up period of more than 2 decades suggesting that depression may be one of the earliest prodromal symptoms of PD (Gustafsson et al., 2015). Despite the ample evidence suggesting that depression is a risk factor for PD, there is still debate as to whether this relationship is unidirectional (Leentjens, 2015) since other investigators suggest that PD may be a risk factor for depression (Reijnders et al., 2008).

**Table 2:** Studies investigating the association between depression and Parkinson’s disease.

Study	Study design	Main results
Shiba et al. (2000)	Prospective, case-control, USA	Frequency of depression is higher in PD than controls. Depression may precede PD motor symptoms.
Schuurman et al. (2002)	Retrospective, case-control, Netherlands	Strong positive association between depression and subsequent incidence of PD.
Leentjens et al. (2003b)	Prospective, case-control, Netherlands	Depression precedes PD. The average time-span between the first episode of depression and the diagnosis of PD was 10 years.
Fang et al. (2010)	Case-control, USA	Positive association between depression and a higher subsequent risk of PD. Depression was detected more than 15 years before the diagnosis of PD.
Jacob et al. (2010)	Retrospective, case-control, USA	Positive association between depression and subsequent risk of PD in men but not women.
Shen et al. (2013)	Retrospective, case-control	Patients with depression were 3.24 times more likely to develop PD.
Gustafsson et al. (2015)	Prospective, case-control	Positive association between depression and subsequent risk of PD. Depression may predate two decades before onset of PD.

Several hypotheses have been proposed to explain the relationship between depression and PD. One plausible explanation is that depression in PD represents a psychological response to the limitations and disabilities imposed with a diagnosis of PD (Mindham, 1970). However, this hypothesis does not explain cases where depression precedes PD. Other hypotheses have provided more meaningful biological explanations for the relationship between depression and PD. For instance, the “serotonin hypothesis” is based upon the finding that serotonin activity is lower in the brains of patients with depression and PD compared to healthy individuals (Mayeux, 1990). Brain lesions in the orbital frontal cortex and basal ganglia are found in patients with PD and depression (Gareri et al., 2002). Another explanation is the Braak hypothesis, which explains the sequential deposition of SNCA starting in the olfactory tract and lower brainstem regions and proceeding upwards to different parts of the brain (Braak et al., 2003). The sequential accumulation of SNCA, divided in six Braak stages (I-VI) is a gold standard metric to classify the degree of pathology in PD (Braak et al., 2003). According to the Braak hypothesis the raphe nuclei found in the brainstem, whose main function is to release serotonin, is affected in Braak stage 2; whereas the substantia nigra, which plays an important role in motor control, is affected later in Braak stage 3 of the disease. This finding may provide a pathophysiological explanation as to how depression can later develop into PD (Leentjens, 2015). It may also suggest that PD and depression are linked by a common pathophysiological process (Leentjens, 2015).

Inflammation has been widely implicated in both depression and neurodegeneration. Proinflammatory cytokines are documented to cause alterations in serotonin and dopamine neurotransmission leading to depression and PD (Pessoa Rocha et al., 2014). In this context, persistent inflammation, which is widely documented in PD, accompanied with high levels of inflammatory marker C-reactive protein have been found in the brain of depressed patients (Felger et al., 2016). Nonetheless, the precise mechanism underlying the association between depression and PD remains unknown.

Only a few studies have investigated shared genetic risk factors between PD and depression. Given that serotonin transport is compromised in PD and depression, it has been suggested that both diseases may share a genetic susceptibility *via* the serotonergic system. Allelic variations in the serotonin transporter *5-HTTLPR* have been suggested to be a risk factor for depression in PD patients, but the evidence has been inconsistent across studies (Mössner et al., 2001; Burn et al., 2006; Gao and Gao, 2014). One study suggested that depression is more common in PD patients carrying a G2019S-*LRRK2* mutation than in non-carriers (Belarbi et al., 2010). Collectively, these studies indicate that shared genetic risk factors between PD and depression are poorly understood. Future genome-wide association studies assessing gene pleiotropy between PD and depression will be helpful to clarify the genetic relationship between both diseases.

Drugs that enhance dopamine neurotransmission may be useful therapeutics for patients with treatment-resistant depression (Dunlop and Nemeroff, 2007). For example, monoamine oxidase inhibitors (MAOI), which enhance dopamine function, have shown positive effects in patients with treatment-resistant depression (Fawcett et al., 2016). Monoamine oxidase type B (MAOB) inhibitors have elicited neuroprotective effects in preclinical models of PD and longer exposure to these inhibitors have been associated with less clinical decline in PD patients (Hauser et al., 2017) and reduced risk of dyskinesia (Dashtipour et al., 2015). Early treatment with rasagline, another MAOB inhibitor, resulted in improvement in UPDRS scores thus showing promise as a neuroprotective agent in PD patients (Olanow et al., 2009). However, treatment of rasagline at higher doses did not show the same neuroprotective effects. Other promising antidepressant drugs for PD tested in placebo-controlled studies are summarized in detail elsewhere (Aarsland et al., 2011).

Successful therapeutic intervention in patients with depression relies upon accurate diagnosis. Depression is currently diagnosed based on self-report and there is discrepancy between the severity of patient’s self-reported depression and the clinician’s rated depression symptoms. Also important is the inability of patients to adequately characterize their symptoms and the differences between depression scoring systems (Riedel et al., 2010). A reliable diagnosis of depression and PD is challenging. There is a prominent overlap in symptoms between both diseases. For example, fatigue, loss of energy, psychomotor retardation, anemia, slowing of intellectual functions, concentration difficulties, reduced appetite and insomnia can be observed in both depression and PD patients. In addition, the different methods to assess depression in PD patients could also explain the variations observed in the literature (Starkstein et al., 1990; Leentjens et al., 2003a; Assogna et al., 2013). Of note, Hoogendijk et al. (1998) conducted a study with PD patients and rated the presence of depressive symptoms using both “inclusive” and “exclusive” methods. Whereas inclusive approach considers all symptoms as related to depression regardless of their overlap with PD or other medical conditions, the exclusive approach does not consider any overlapping symptoms as counting toward symptom severity or diagnostic criteria (Hoogendijk et al., 1998). Using the inclusive approach, depression was diagnosed in 23% of patients, but the frequency was reduced to 13% when it had been used the exclusive approach (Hoogendijk et al., 1998). As a result, depression is largely under-recognized and misdiagnosed in patients. As in PD, there are no fully validated biomarkers for depression. Although several molecular signatures have been identified in blood of patients with depression (Redei et al., 2014; Bilello et al., 2015), diagnosis of depression in PD patients remains challenging. An easily accessible biomarker that could inform about comorbid depression in PD would be useful to provide individualized treatment of patients. In this regard, a recent study identified that protein levels of S100A10 were associated with depression scores in PD patients with depression compared to those without Green et al. (2017). Similarly, serum levels of BDNF were lower in PD patients with depression than those without depression (Wang et al., 2017). Integrative meta-analysis of transcriptomic studies in drug-naïve PD and depression studies identified shared molecular networks in both diseases. Expression levels of nicotinamide phosphoribosyltransferase (*NAMPT*) mRNA, the most highly ranked gene in the meta-analysis, was upregulated in drug-naïve PD patients compared to healthy controls (Santiago et al., 2016). Yet, it remains unclear if *NAMPT* mRNA will be a useful biomarker for depression in PD patients. Nonetheless, combination of these biomarkers with other clinical information could be key to advance individualized treatment of PD patients with depression.

## Anemia and Parkinson’s Disease

Anemia, a condition characterized by reduced levels of hemoglobin and systemic iron stores, has been also associated with PD. Globally, anemia affects 1.62 billion people and it is more prevalent in women and young children. Anemia is diagnosed using a blood test to determine the number of red blood cells and hemoglobin levels. Hemoglobin levels of <13 g/dL in men or 12 g/dL in women are characteristic of anemia. There are several types of anemia including some hereditary forms but the most common type is iron deficiency anemia.

Epidemiological studies have suggested an association between anemia and PD (Table 3). One of the first studies found a higher risk of PD among men who reported multiple blood donations (Logroscino et al., 2006). This study prompted further investigations in the possible role of anemia as a risk factor for PD. Another study suggested that anemia might precede motor symptoms by 20 years (Savica et al., 2009). In this study, individuals who later developed PD displayed a downward shift of hemoglobin levels compared to controls as early as 20–29 years before the onset of PD. These findings are supported by recent studies in different populations. For instance, lower levels of hemoglobin associated with disease severity in PD patients (Deng et al., 2017). Recently, a positive association between anemia and PD was found in a large study of 86,334 newly diagnosed anemic patients. This study suggested that *de novo* anemic patients might develop PD four or more years after the initial diagnosis of anemia (Hong et al., 2016). In this study, patients with iron deficiency anemia exhibited a higher risk of PD independent of iron supplementation and 75.9% of all the anemic patients were women. This is not surprising since the prevalence of anemia is higher in non-pregnant woman (30.2%) than in men (12.7%) according to the World Health Organization^3^. On the contrary, PD is more prevalent in men than in woman (de Lau and Breteler, 2006; Ascherio and Schwarzschild, 2016). Despite these differences, epidemiological studies have not identified any sex-specific factors in the risk of developing PD among anemic patients. In fact, the association between anemia and PD has remained significant after adjusting for hysterectomy, a condition that has been associated with an increased risk of PD in women (Benedetti et al., 2001). Addressing sex specific differences regarding the risk of developing PD among anemic patients is warranted. Other neurological diseases including AD (Pandav et al., 2004; Faux et al., 2014), cognitive decline (Peters et al., 2008), and restless leg syndrome (Satija and Ondo, 2008; Piao et al., 2017) have been also associated with anemia. Therefore, the potential mechanisms linking anemia and neurodegeneration merits further investigation.

**Table 3:** Studies investigating the association between anemia and Parkinson’s disease.

Study	Study design	Main results
Logroscino et al. (2006)	Prospective, case-control, USA	Multiple blood donations are associated with an increased risk of PD in men.
Savica et al. (2009)	Retrospective, case-control, USA	Anemia is associated with an increased risk of PD.
Hong et al. (2016)	Retrospective, case-control, Taiwan	Anemia is associated with an increased risk of PD. PD might develop 4 years or more after the initial diagnosis of anemia.
Deng et al. (2017)	Case-control, China	Hemoglobin levels are lower in PD patients and are associated with disease severity.

The presence of anemia in PD could be also an indicator of vitamin B12 deficiency (Madenci et al., 2012) or poor absorption of other nutrients (Logroscino et al., 2006). This is not surprising since it is well documented that nutrition plays a crucial role in PD (Seidl et al., 2014). Dysregulated iron metabolism is another plausible explanation linking both diseases. Disrupted iron metabolism has been extensively implicated in the pathogenesis of PD. It has been documented that PD patients exhibit lower serum iron levels (Savica et al., 2009; Pichler et al., 2013; Medeiros et al., 2016). Interestingly, late stage PD patients had lower levels of iron, ferritin and total iron binding capacity suggesting that markers of disrupted iron metabolism could be useful markers for disease stratification in PD (Deng et al., 2017). A recent transcriptomic meta-analysis identified a significant downregulation of genes associated with hemoglobin and iron metabolism including, hemoglobin delta (*HBD*), alpha hemoglobin stabilizing protein (*ASHP*) and solute carrier family 11 member 2 (*SLC11A2*) in blood of PD, thus reinforcing the relevance of these pathways in the disease pathogenesis (Santiago and Potashkin, 2017). Additionally, eryptosis, a process characterized by the shrinkage and death of red blood cells, has been observed in both anemia and PD (Pretorius et al., 2014). Interestingly, treatment with erythropoietin, a hormone that promotes the formation of red blood cells, elicited neuroprotective effects in preclinical models of PD (Farmer et al., 2015; Jang et al., 2016; Carelli et al., 2017) and clinical trials demonstrated its safety, tolerability and efficacy in PD patients (Pedroso et al., 2012; Jang et al., 2014). However, treatment with erythropoietin has shown beneficial effects only in non-motor symptoms in PD patients (Jang et al., 2014). Future larger randomized and placebo-controlled clinical trials are needed to evaluate the potential neuroprotective properties of erythropoietin in PD patients.

**Table 4:** Studies investigating the association between melanoma and Parkinson’s disease.

Study	Study design	Main results
Olsen et al. (2005)	Retrospective, Cohort study, Danish cancer registry	Increased relative risks of melanoma and breast cancer in PD patients.
Olsen et al. (2006)	Retrospective, Case-control, National Danish Hospital Register	Increased prevalence of melanoma before the diagnosis of PD.
Baade et al. (2007)	Australian	Melanoma patients had a 3-fold increased risk of dying from PD.
Inzelberg and Israeli-Korn (2009)	Pubmed search of cohort studies	The increased risk of melanoma for PD patients cannot be attributed to L-dopa treatment.
Bertoni et al. (2010)	Prospective clinicopathological study, USA cohort.	Melanoma prevalence is higher in PD patients.
Liu et al. (2011)	Meta-analysis	Melanoma occurrence is higher after the diagnosis PD, but not before PD diagnosis.
Rugbjerg et al. (2012)	Cohort study, National Danish Hospital Register	Increased risks for malignant melanoma, nonmelanoma skin cancer and breast cancer in PD patients.
Kareus et al. (2012)	Cohort study, Population-based pedigree-linked study, Utah cancer registry	Risk association for melanoma in PD patients as well as increased risk for PD in relatives of individuals with melanoma. Association between PD and prostate cancer.
Wirdefeldt et al. (2014)	Cohort study, Swedish Multi-Generation Register	Melanoma risk is higher among PD patients. Familial mechanisms do not explain the association.
Ong et al. (2014)	Cohort study, All-England record-linked hospital and mortality dataset	Increased risk of melanoma, breast, uterine and renal cancers in PD.
Constantinescu et al. (2014)	Cohort study, National Institutes of Health (NIH) Exploratory Trials in PD (NET-PD) LS-1	The risk for developing melanoma was higher than expected in the NET-PD LS-1 cohort compared with the general population.
Peretz et al. (2016)	Retrospective, Cohort study, Israel	No difference in the risk of any type of cancer among PD patients.

## Cancer and Parkinson’s Disease

Several epidemiological studies have reported an association between cancer and PD, supporting generally a decreased risk of PD among almost all cancer types. A meta-analysis of 29 studies found that a diagnosis of PD was associated with an overall 27% decreased risk of cancer, and 38% decreased risk after excluding melanoma and other skin tumors (Bajaj et al., 2010). Consistently, another meta-analysis of 50 observational studies reported a 17% decreased risk of cancer in PD patients (Catalá-López et al., 2014). Cancers of the prostate, lung, bladder, colorectal, blood and uterus were among the most reduced in PD patients (Feng et al., 2015). A detailed review of associations between PD and cancer has been published elsewhere (Feng et al., 2015).

While most of the studies suggest an overall negative association between PD and cancer, some studies have indicated the opposite. For example, several studies have reported that PD patients are at higher risk of developing brain tumors (Lin et al., 2015; Tang et al., 2016; Ye et al., 2016) and breast cancer in women (Olsen et al., 2005; Rugbjerg et al., 2012). Additionally, PD patients harboring a G2019S *LRRK2* mutation have been shown to have higher cancer rates than non-mutation carriers, especially for hormonal-related cancers and breast cancer in women (Agalliu et al., 2015). Conversely, some studies have not found a significant association between breast cancer and PD (Elbaz et al., 2002; Lo et al., 2010; Lin et al., 2015).

Interestingly, melanoma is the only cancer type for which a connection with PD has been well documented (Inzelberg and Israeli-Korn, 2009; Ferreira et al., 2010; Liu et al., 2011; Inzelberg et al., 2016a; Shalaby and Louis, 2016; Dalvin et al., 2017). Several studies have demonstrated that PD patients have a higher risk of developing melanoma and vice versa (Olsen et al., 2005, 2006; Baade et al., 2007; Inzelberg and Israeli-Korn, 2009; Bertoni et al., 2010; Liu et al., 2011; Kareus et al., 2012; Rugbjerg et al., 2012; Constantinescu et al., 2014; Ong et al., 2014; Wirdefeldt et al., 2014; Peretz et al., 2016; Table 4). Epidemiological evidence has consistently reported a positive association between melanoma and PD (Feng et al., 2015). Nonetheless, the relative risk of melanoma among PD patients has a considerable variability ranging from 0.5 to 20.9 (Feng et al., 2015). Some of the variabilities in these studies may arise from biases due to inclusion/exclusion criteria, sample size, genetics and other confounding variables.

Impaired autophagy, dysfunction of melanin-related enzymes and genetic risk factors have been proposed to be causative factors, but a single underlying mechanism for the linkage between melanoma and PD remains unknown (Inzelberg et al., 2016a). Several interesting genetic links have been identified between melanoma and PD. Earlier studies reported that individuals with first-degree family history of melanoma had a 85% increased risk of PD after adjusting for smoking, caffeine intake, and ethnicity (Gao et al., 2009a). Interestingly, dysregulation of genes involved in melanin synthesis have been suggested as a potential genetic intersection between melanoma and PD. For instance, SNCA is highly expressed in malignant and benign melanoma and it interacts with enzymes involved in melanin synthesis (Matsuo and Kamitani, 2010; Pan et al., 2012). Several mutations in melanocortin 1 receptor (*MC1R*), a key gene involved in human pigmentation, have been associated with risk of PD in several populations. A large case-control study including more than 120,000 US men and women reported that red hair and the associated *MC1R* p.R151C polymorphism, both of which confer high melanoma risk, were associated with greater risk of PD (Gao et al., 2009b). Interestingly, PD risk increased with increasing lightness of hair color, in particular for young onset PD. Other case-control studies found that a different *MC1R* variant p.R160W was associated with an increased risk of PD in a Spanish population (Tell-Marti et al., 2015) but not in a Chinese population (Shi et al., 2016). Consistent with these findings, a recent meta-analysis found that red hair and *MC1R p.R151C* variant, but not the p.R160W variant, were associated with a greater risk of PD (Chen et al., 2017b).

These genetic studies prompted investigations into the molecular mechanisms underlying the linkage between melanoma and PD. In this context, inactivation of *MC1R* in mice resulted in increased vulnerability of dopaminergic cells to 6-hydroxydopamine (6-OHDA) and 1-methyl-4-phenyl-1,2,3,6-tetrahydropyridine (MPTP) toxins (Chen et al., 2017a). Strikingly, treatment with a MC1R agonist conferred neuroprotection against MPTP-induced toxicity thus suggesting MC1R as a potential therapeutic target.

Several PD genes have been shown to play a role in oncogenesis. For example, dysregulation of *PARK1* and *PARK4*, associated with some familial forms of PD, are observed in cancers such as adenocarcinoma, lung, colorectal, brain, melanoma, prostate and non-Hodgkin lymphomas (Fung et al., 2003; Matsuo and Kamitani, 2010; Bethge et al., 2014; Li et al., 2015). Similarly, genetic alterations in *PARK2*, also linked to familial PD, have been observed in glioblastoma, lung, colorectal, renal cell carcinoma, melanoma and pancreatic cancers (Veeriah et al., 2010; Gong et al., 2014, 2017; Hu et al., 2015; Inzelberg et al., 2016b). PINK1 (PARK6) a kinase involved in the regulation of autophagy and the cell cycle, has been involved in glioblastoma, and ovarian cancers (Berthier et al., 2011; Devine et al., 2011; O’Flanagan et al., 2016). Recently, PINK1 expression has been associated with poor response to chemotherapy (Yamashita et al., 2017). Mutations in DJ1 (PARK7, a regulator of the tumor suppressor PTEN) is observed in PD patients and in breast, lung, pancreatic, gastric and prostate cancers (Hod, 2004; He et al., 2011; Zeng et al., 2011; Kawate et al., 2013; Li et al., 2013). Another example, *LRRK2* is overexpressed in papillary renal and thyroid carcinomas (Looyenga et al., 2011) and mutations in *LRRK2* are associated with an increased risk for breast, non-skin and hematological cancers (Inzelberg et al., 2012; Ruiz-Martínez et al., 2014; Agalliu et al., 2015).

Several cancer genes have also been shown to play a role in the development of PD. For example, the tumor suppressor p53 activates the *SNCA* promoter (Duplan et al., 2016) whereas the proto-oncogene tyrosine kinase c-Abl regulates the degradation of SNCA (Mahul-Mellier et al., 2014). Interestingly, treatment with nilotinib, a c-Abl inhibitor, prevented the loss of dopaminergic neurons in MPTP-treated mice (Karuppagounder et al., 2014), thus demonstrating its potential as a therapeutic target for PD. Furthermore, a small clinical trial using nilotinib demonstrated its safety, tolerability and suggested a possible beneficial effect in motor and cognitive outcomes in 12 subjects with PD (Pagan et al., 2016). Nonetheless, given the small sample size, larger randomized, double blind and placebo-controlled clinical trials are needed to evaluate its therapeutic potential.

## System-Level Understanding of Parkinson’s Disease

The molecular underpinnings in PD remain largely elusive, as genetic risk factors only explain a small fraction of the cases. The increasing number of studies suggesting that other diseases including diabetes, depression, anemia and cancer may be associated with PD strengthens the importance of a system-level understanding of PD and its comorbidities. In this context, several system-biology approaches, in particular, network-based approaches, have uncovered shared genetic associations, mechanisms, biomarkers and therapeutics for PD (Santiago and Potashkin, 2014a; Santiago et al., 2017). Network analyses identified shared molecular networks between diabetes and PD. These network analyses uncovered two promising blood biomarkers *APP* and *SOD2* mRNAs, for identifying early stage PD patients (Santiago and Potashkin, 2013a; Santiago et al., 2014). Furthermore, a network-based meta-analysis identified *HNF4A* and *PTBP1*, both associated with diabetes, as potential progression biomarkers for PD (Santiago and Potashkin, 2015b). Although these markers were not differentially expressed in samples obtained from drug-naïve PD patients (Santiago and Potashkin, 2015a), they may be useful prognostic and/or diagnostic markers of PD patients with comorbid diabetes. Given that inflammatory markers are dysregulated in PD and diabetes, levels of TNF, IFNy and myeloid dendritic cells could be potential diagnostic biomarkers for PD (Ciaramella et al., 2013; Eidson et al., 2017). Furthermore, these markers may be valuable to track the response of PD patients to treatment in clinical trials testing antidiabetic drugs. Future larger and well-characterized cohorts including PD patients with impaired glucose metabolism will be crucial to determine the utility of these biomarkers.

In addition to diabetes, analysis of the blood transcriptome has revealed interesting PD links with depression and iron metabolism. For example, transcriptomic meta-analysis and network analysis of blood microarrays from drug-naive patients with PD and depression identified shared genes enriched in pathways related to the immune system, metabolism of lipids, glucose, fatty acids, nicotinamide, lysosome, insulin signaling and type 1 diabetes (Santiago et al., 2016). Among these genes, *NAMPT* was identified as the most dysregulated gene between PD and depression. Combination of *NAMPT* with the University of Pennsylvania Smell Identification Test (UPSIT) was capable to distinguish untreated PD patients from healthy controls with an overall diagnostic accuracy of 86%. Interestingly, pharmacological intervention with a highly specific NAMPT inhibitor conferred neuroprotection in a 6-hydroxydopamine (OHDA) *in vitro* model of PD (Zou et al., 2016). Similarly, transcriptomic meta-analysis of several microarrays identified several genes related to hemoglobin and disrupted iron metabolism, characteristic features of anemia, in blood of PD (Santiago and Potashkin, 2017). In this regard, iron specific genetic variants in hemochromatosis (*HFE*; Guerreiro et al., 2006), transferrin (*TF*; Rhodes et al., 2014) and ceruplasmin (*CP*; Hochstrasser et al., 2005; Mariani et al., 2013) genes have been suggested to play a role in the etiology of PD thus offering additional biomarkers for PD.

Network analyses have been key to identify potential therapeutic targets for PD. For example, network analysis combining genetic and toxicogenomic information identified alvespimycin as a potential neuroprotective agent in PD (Gao et al., 2014). Treatment with alvespimycin attenuated rotenone-induced toxicity *in vitro*. Similarly, construction of protein-protein interaction networks modulating the disruption of autophagy and mitochondrial machinery revealed a network of proteins including p62, GABARAP, GBRL1 and GBRL2 that rescued 1-methyl-4-phenylpyridinium (MPP) induced-toxicity (Keane et al., 2015). Notably, overexpression of these proteins combined, but not each one alone, conferred neuroprotection. Recently, a system-level approach using genome wide analysis and functional gene networks identified 17 shared loci between PD and seven autoimmune diseases including celiac disease, rheumatoid arthritis, type 1 diabetes, multiple sclerosis, psoriasis, ulcerative colitis and Crohn’s disease (Witoelar et al., 2017). Among these autoimmune diseases, the strongest pleiotropic enrichment was observed between PD and Crohn’s disease. The linkage between PD and Crohn’s disease is interesting in light of the studies that suggest that gastrointestinal tract dysfunction may precede the onset of PD (Svensson et al., 2015; Sampson et al., 2016; Liu et al., 2017). Collectively, these studies highlight the potential of unbiased network approaches to identify biomarkers, therapeutics, and shared genetic risk factors between PD and other diseases.

## Personalized Medicine in Parkinson’s Disease

Past clinical trials investigating drugs and neuroprotective agents for PD have largely failed to achieve disease modification in part due to the inadequate definition of PD (Espay et al., 2017). The considerable variability in genetic risk factors, clinical symptoms, disease progression, and treatment response among PD patients have prompted new research efforts to define new clinical subtypes for PD (Berg et al., 2014). Identification of clinical subtypes is expected to facilitate the design of clinical trials, understand disease mechanisms and accelerate personalized medicine. However, no subtypes of PD have been formally introduced or incorporated into current research protocols (Berg et al., 2014). Two examples, onset age and tremor dominance have been proposed as PD subtypes. For example, age is considered because young onset patients have a more robust response to levodopa treatment and fewer abnormalities in cognition whereas tremor predominant subtypes have better prognosis and fewer cognitive disturbances (Berg et al., 2014). Nonetheless, the impact of these subtypes is a topic of debate because some studies have shown that tremor has little effect on prognosis and there is a high misdiagnosis between PD and essential tremor (Selikhova et al., 2009). Other suggested subcategories of PD include prodromal features including hyposmia and RBD (Berg et al., 2014). More recently, three newly PD subtypes were identified using both clinical and molecular data extracted at baseline from early stage drug-naïve PD patients from the Parkinson’s Progressive Markers Initiative (PPMI; Fereshtehnejad et al., 2017). These three distinct subtypes of PD were classified as “mild motor predominant”, “diffuse malignant” and “intermediate”. Interestingly, patients classified as “diffuse malignant” progressed faster with greater decline in cognitive function and dopamine integrity. In the context of biomarkers, “diffuse malignant” subtype had the lowest level of amyloid beta and amyloid-beta total tau ratio in cerebrospinal fluid. It is plausible to hypothesize that these different PD subtypes will respond differently to treatment. Therefore, these results strengthen the importance of identifying and formally defining new clinical subtypes of PD in order to accelerate individualized treatment.

**Figure 2:**
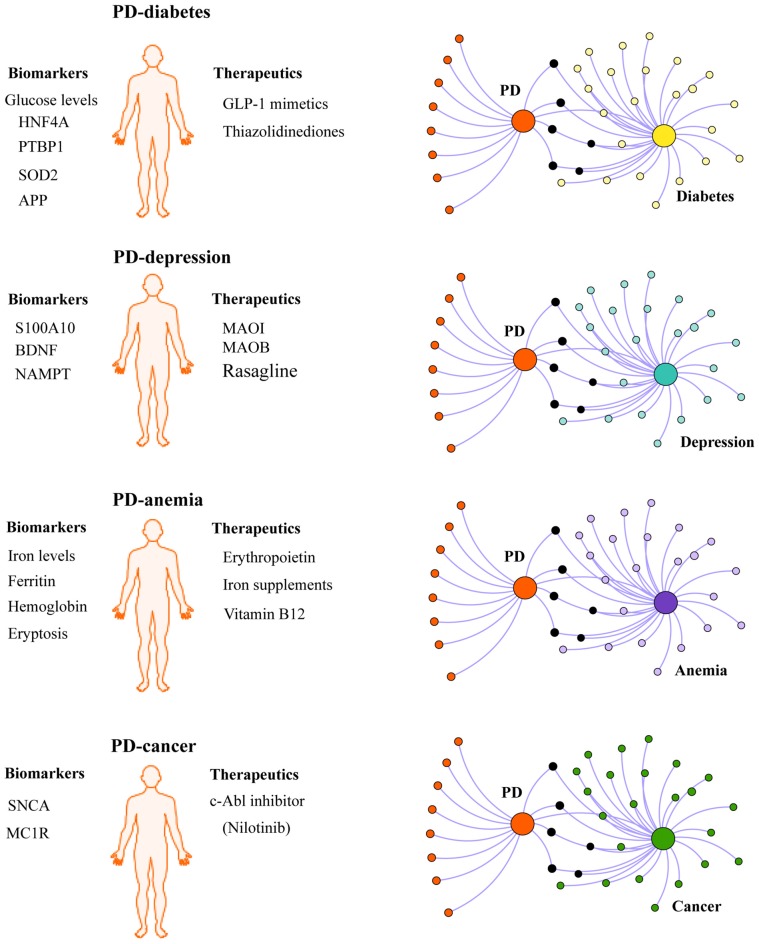
Integrating PD comorbidities in personalized medicine. Understanding disease comorbidities in PD is expected to advance individualized treatment for PD patients. For example, PD patients with comorbid diabetes may respond better to antidiabetic drugs currently under investigation in clinical trials for PD (GLP-1 mimetics and thiazolidinediones). Similarly, disease-modifying effects may be observed in PD patients with other comorbidites recruited for clinical trials testing other potential drugs including monoamine oxidase inhibitors (MAOI), monoamine oxidase type B inhibitors (MAOB, Rasagline), erythropoietin and c-*Abl* inhibitors (Nilotinib). In the context of biomarkers, some biomarkers may be more useful to diagnose PD patients with comorbid depression, diabetes, cancer or anemia, than other patients with different clinical subtypes of PD. Network-based approaches can be exploited to investigate the molecular mechanisms linking PD with comorbidities and to identify biologically relevant biomarkers and potential therapeutic targets.

Given the influence of comorbidities in the disease pathogenesis and progression, we propose that chronic comorbidities such as diabetes and depression should also be taken into consideration when defining PD subtypes (Figure 2). For instance, it is conceivable that PD patients with comorbid diabetes may respond better to insulin sensitizing drugs being tested in clinical trials than non-diabetic PD patients. In the context of diagnosis, some biomarkers may be better suited for identifying PD patients with comorbid diabetes or depression. In this regard, biomarkers identified in shared pathways between PD and diabetes (Santiago and Potashkin, 2013a, 2015b; Santiago et al., 2014), inflammation (Ciaramella et al., 2013; Eidson et al., 2017) or PD and depression (Santiago et al., 2016; Green et al., 2017; Wang et al., 2017) could be useful diagnostic tools. More recently, an unbiased chemical screen identified the β2-adrenoreceptor (β2AR) as a regulator of *SNCA* (Mittal et al., 2017). This study uncovered that the β2AR agonist salbutamol, a drug commonly prescribed to treat asthma, was associated with a reduced risk of developing PD in a Norwegian population. Although a connection between allergic diseases and PD is rare, a cross-sectional retrospective study revealed that patients with asthma had an increased risk of PD in a Taiwanese population (Cheng et al., 2015). The relationship between asthma and PD remains unknown. Future network-based analyses will be helpful to understand the molecular mechanisms underlying the connection between these diseases and may reveal additional biomarkers and therapeutic targets. A phenotypic-driven approach to biomarker discovery has been recently proposed (Espay et al., 2017). Therefore, a system-level understanding of PD and its associated comorbidities is expected to help identify other PD subtypes and advance individualized treatment.

## Concluding Remarks

Collectively, comorbidities have important implications in the health outcomes and clinical management of PD patients. Increasing evidence from epidemiological studies suggest that diabetes, depression and anemia may appear before the onset of PD thus highlighting the importance of recognizing these comorbidities as potential risk factors for PD. Notably, understanding the molecular and clinical evidence of comorbidities in PD has paved the way for the discovery of novel therapeutic strategies for PD. In this context, common drugs to treat diabetes and cancer are now being tested in clinical trials for PD. In parallel, emerging “big-data” and system-biology approaches are helping in the discovery of biomarkers, therapeutic targets and understanding the molecular mechanisms underlying the occurrence of these comorbidities in PD. We expect that a multidimensional approach to PD incorporating comorbidities will provide new venues for the advancement of individualized treatment in PD patients.

## Author Contributions

JAS and VB wrote the first draft of the manuscript. JAS, VB and JAP edited and reviewed the final draft of the manuscript.

## Conflict of Interest Statement

The authors declare that the research was conducted in the absence of any commercial or financial relationships that could be construed as a potential conflict of interest.

## Footnotes

^1^http://www.parkinsonassociation.org/facts-about-parkinsons-disease/

^2^https://www.nimh.nih.gov/health/statistics/prevalence/major-depression-among-adults.shtml

^3^http://www.who.int/en/
